# Detecting Phase-Synchrony Connectivity Anomalies in EEG Signals. Application to Dyslexia Diagnosis

**DOI:** 10.3390/s21217061

**Published:** 2021-10-25

**Authors:** Marco A. Formoso, Andrés Ortiz, Francisco J. Martinez-Murcia, Nicolás Gallego, Juan L. Luque

**Affiliations:** 1 Communications Engineering Department, University of Málaga, 29071 Málaga, Spain; marco.a.formoso@ic.uma.es (M.A.F.); njgm@ic.uma.es (N.G.); 2 Andalusian Research Institute in Data Science and Computational Intelligence (DaSCI), 18014 Granada, Spain; fjesusmartinez@ugr.es; 3 Department of Signal Theory, Networking and Communications, University of Granada, 18014 Granada, Spain; 4 Department of Basic Psychology, University of Malaga, 29019 Málaga, Spain; juanluque@uma.es

**Keywords:** functional connectivity, EEG, anomaly detection, self-organizing maps, phase locking value, circular correlation

## Abstract

Objective Dyslexia diagnosis is a challenging task, since traditional diagnosis methods are not based on biological markers but on behavioural tests. Although dyslexia diagnosis has been addressed by these tests in clinical practice, it is difficult to extract information about the brain processes involved in the different tasks and, then, to go deeper into its biological basis. Thus, the use of biomarkers can contribute not only to the diagnosis but also to a better understanding of specific learning disorders such as dyslexia. In this work, we use Electroencephalography (EEG) signals to discover differences among controls and dyslexic subjects using signal processing and artificial intelligence techniques. Specifically, we measure phase synchronization among channels, to reveal the functional brain network activated during auditory processing. On the other hand, to explore synchronicity patterns risen by low-level auditory processing, we used specific stimuli consisting in band-limited white noise, modulated in amplitude at different frequencies. The differential information contained in the functional (i.e., synchronization) network has been processed by an anomaly detection system that addresses the problem of subjects variability by an outlier-detection method based on vector quantization. The results, obtained for 7 years-old children, show that the proposed method constitutes an useful tool for clinical use, with the area under ROC curve (AUC) values up to 0.95 in differential diagnosis tasks.

## 1. Introduction

Electroencephalography (EEG) is a non-invasive way to obtain information about brain activity. It has been widely used with different objectives, from the study of cortical brain activity related to the cognitive development under specific tasks [1], to the exploration of the brain processes involved in neurological diseases such as Alzheimer’s disease [2,3], Parkinsonian syndromes [4,5], epileptic disorders [6,7,8], and other psychiatric disorders such as schizophrenia [9]. Moreover, EEG has been widely used in experimental neuropsychology in the quest for answers about how the brain processes different stimuli and the cortex areas involved. This is the case of learning difficulties, since their neural origin remain unknown. Furthermore, efforts to explain the brain processes involved in language processing constitute a way for the early diagnosis of these disorders.

One of the most common learning disorders is Developmental Dyslexia (DD), a specific difficulty in the acquisition of reading and writing skills not related to mental age or inadequate schooling. DD may severely affect the self-esteem of affected children and may determine school failure. Its prevalence is estimated between 5% and 12% of the population [10], depending on the reading performance benchmark. DD diagnosis is usually carried out by behavioural tests that can only be performed by readers, whose results depend on the children mood and the issuance of a diagnostic report requires a detailed analysis of the results by expert clinicians. On the other hand, the early diagnosis of DD plays a major role for the success of specific intervention tasks developed to mitigate the consequences of DD. Then, diagnosis techniques based on the extraction of biological markers provide valuable and objective information for the clinicians and pave the way for the early diagnosis, facilitating the application of an intervention program in pre-readers [11]. In fact, neurophysiological signals constitute an objective way to evaluate neurological or behavioural disorders that are usually subjectively assessed by clinicians.

Brain functional connectivity refers to the coordinated activation of brain areas arising when the subject performs a specific task or staying in resting state. Indeed, some previous works have developed different methods to estimate the functional connectivity by searching for patterns that characterize different neurological diseases such as Alzheimer’s or Parkinson’s Diseases using functional imaging techniques [12,13,14,15], EEG [16,17], or classify different sleep stages [18]. Moreover, the use of brain connectivity has also been used in cognitive neuroscience to identify brain areas involved in language and learning tasks [19,20]. The use of EEG to explore functional connectivity provides opportunities due to its high temporal resolution, allowing researchers to measure the synchronization between brain areas. That synchronization can be interpreted as cooperation from the functional connectivity point of view, and inferred from the phase difference between EEG channels, the statistical relationship between different EEG frequency bands (namely, Cross-Frequency Coupling), or using techniques that figure out inter-channel causality [21].

In this line, some previous works used EEG and Magnetoencephalography (MEG) to explore the basis of DD using speech-based stimuli, under the assumption that DD is characterized by reduced awareness of speech units [22]. Hence, atypical neural entrainment at different rates may arise in affected subjects. Other works in the search of the basis of speech coding, hypothesize that DD originates from the atypical synchronization in the right hemisphere to the lower EEG bands [23,24]. In [25] differences between dyslexic and control groups were found in the preferred phase of entrainment of Delta band after presenting a visual and auditory stimulus to the child.

An example of EEG classification where an auditory stimulus is used can be found in [26]. In this work, Gibbon et al. presented either a drumbeat or a syllable to 8-week-old infants while recording the EEG. They were able to classify whether the EEG correspond to the drumbeat or the syllable with an AUC of 0.875 for a convolutional neural network and an AUC of 0.86 for a support vector machine. Changing the task while recording the EEG from a hearing to writing, Perera et al. [27] were able to detect dyslexic children using a support vector machine. They recorded EEGs while the subjects were doing a writing task (pen and paper) and typing task (on a keyboard). Besides using all the channels at once to classify, they selected the channels distinguishing several brain parts obtaining different results for each one. Moreover, in [28], several stimuli at different frequencies were shown to the children. Then, temporal and spectral features from the EEGs at different frequency were used to build the feature space, obtaining AUC values ranging from 0.69 to 0.89. On the other hand, [29] uses periodogram-based features. Specifically, the spectral density is obtained from the EEGs and then Principal Component Analysis is used to reduce the dimension of the spectral density vector. In order to classify, they use a support vector machine, comparing the classification performance of the whole spectral density vector and the reduce version of it. This method provided AUC values up to 0.75. In [30], power-based connectivity features were computed to train a neural network (denoising autoencoder) to generate features to represent dyslexic and control groups in a lower dimensional space.

As shown in the works cited above, the use of EEG signals in search of descriptive patterns requires the use of specific signal processing techniques due to its low signal-to-noise (SNR) ratio and the presence of multiple artefacts such as ocular, muscular or cardiac. These have to be properly removed without affecting useful information. As a consequence, different preprocessing methods such as Independent Component Analysis (ICA) are commonly used to remove known artefacts by a source separation process.

On the other hand, the feature extraction process has to provide discriminative enough descriptors for modelling the categories present in the dataset. These features can be based on time, frequency [31], or time-frequency [32], that can also be used to derive complex relationships among brain areas. Depending on the specific descriptor, these relationships can be interpreted as *functional connectivity*, as in the case of this work. Connectivity measures can be derived from frequency and phase variations and power spectrum, and they can be split into two types depending on the method used to compute those relationships:

- Methods that characterize the statistical relationships between electrodes but in the same frequency band. In this way, Spectral Coherence (SC) provides a way to measure the synchronization between channels, which may indicate a *connection* from the functional point of view between the neuron clusters in the two areas involved [33]. Other connectivity measures can be computed from the phase angle differences between channels over time [21].
- Methods that characterize the statistical relationships between the activity in two channels at different frequency bands. The measure provided by these methods is commonly referred as *Cross Frequency Coupling* (CFC). Phase-Amplitude Coupling (PAC) is a representative and practical example for computing the CFC which has neural and physical implications [34].

We use SC descriptors to estimate the connectivity between channels in the five EEG bands (Delta, Theta, Alpha, Beta, and Gamma) and then, to extract discriminative features for the differential diagnosis of DD. These EEG were obtained using a novel method based on the application of auditory stimuli at different frequencies that activate the low-level processing network in the brain cortex. Moreover, an anomaly detection approach is implemented using a method that combines unsupervised learning by vector quantization and a Bayesian classifier. The proposed method allows working with not very large databases, overcomes the imbalance problem and reduces the overfitting.

The main aim of the work is twofold: firstly, it aims to demonstrate that low-level auditory processing produces different patterns in the brain networks involved. This connectivity is studied by means of phase synchronization between EEG channels. This methodology is not only relevant for diagnostic purposes but also for the study of the differences in the brain processes developed during auditory processing in controls and dyslexic children, paving the way to go deeper into the biological basis of dyslexia. Secondly, the overall classification pipeline provides a method that can detect dyslexic subjects in an objective way, only using EEG signals. This can be used as an effective tool for clinical practice. The main contributions of this work can be summarized as:

1. We use low level auditory stimuli to study the brain processes involved in language processing, instead of previous works that use only speech-based stimuli [22].
2. Connectivity between brain areas is searched by means of phase synchronization between EEG channels, which is computed using the Circular Correlation.
3. An anomaly detection approach has been implemented using a method that combines unsupervised learning by vector quantization and a Bayesian classifier. The proposed method allows working with not very large databases, overcomes the imbalance problem and reduces the overfitting.

After this introduction, the rest of the paper is organized as follows. Section 2 shows the materials and methods used in this work, including the database and the acquisition protocols used to compose it. Moreover, this section presents the foundations of the methods used to measure the inter channel phase synchrony. Then, Section 4 shows the proposed classification method that considers the non-control samples as anomalies. Section 5 and Section 6 presents the main results obtained with the proposed methodology and the discussion, respectively. Finally, conclusions are drawn in Section 7.

## 2. Materials and Methods

### 2.1. Data Acquisition

EEG data used in this work was provided by the *Leeduca* group at the University of Málaga. Control and experimental groups are extracted by a carefully screening process from a cohort (N = 700) followed from 4 years to the second evaluation of 7 years in 20 different primary schools (Junta de Andalucía). This way, the socioeconomic status (SES) had a longitudinal dynamic evaluation of the subjects, plus ATLAS [35] (A self-report questionnaire on reading-writing difficulties for adults) family risk information, plus a complete report at 7 years old, which included standard assessment tasks. Comorbidities with other neurodevelopmental disorders such as Language Impairment (LI), Speech Sound Disorder (SSD), Attention Deficit Hyperactivity Disorder (ADHD), Autism, and other auditory or visual sensory deficit disorders were taken into account in the screening process, along with information about other relevant conditions which can affect reading achievement, as immigration or bilingualism [36].

In the experiments, EEG signals were recorded by a Brainvision actiCHamp Plus with a 32 channels amplifier that allows a sampling rate up to 500 Hz using active electrodes (actiCAP, Brain Products GmbH, Germany). EEG was recorded during 5-min sessions at a sampling rate of 500 Hz, while presenting an auditory stimulus to the subject. These auditory stimuli consisted on white noise 100% amplitude modulated at 4.8 Hz and 16 Hz. These measures were repeated two times. Stimulus were determined by expert linguistic psychologists studying the main frequency components present in voice, corresponding to syllables and phonemes. The study of the statistical distribution of these components determined a component of 4.8 Hz and 16 Hz for syllables and phonemes, respectively, in Spanish speakers.

Database descriptive statistics are shown in Table 1. Subjects in the database are right-handed, Spanish native speakers, and have neither hearing impairments nor vision problems.

**Figure 1:**
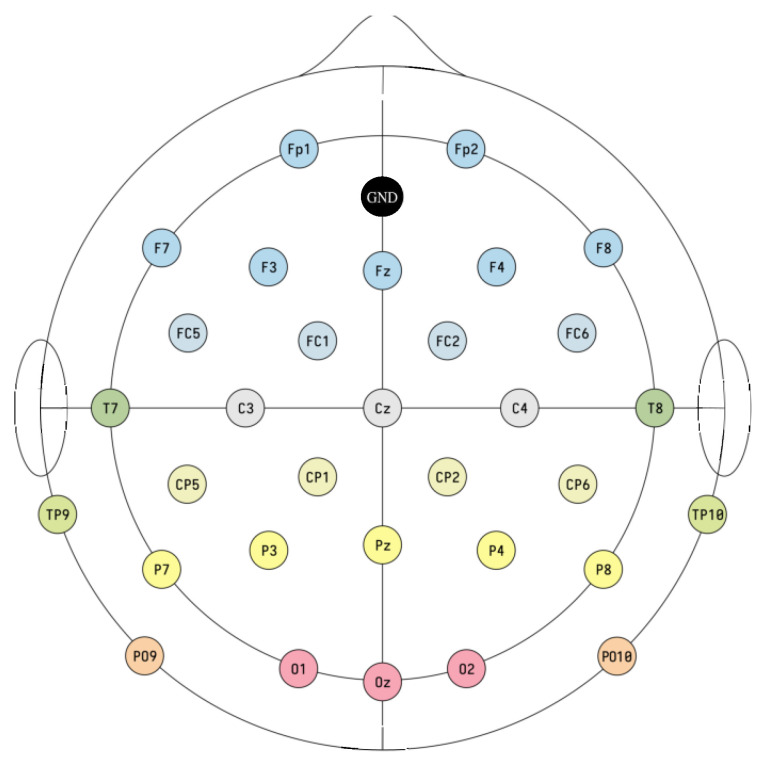
Electrode montage in the extended 10–20 system used in the experiments. All 32 channels plus GND. Cz is used as reference.

The location of 32 electrodes in the 10–20 standardized system used in the experiments is shown in Figure 1.

### 2.2. Data Preprocessing

EEG signals were first preprocessed to remove artefacts related to eye-blinking, and impedance variations due to movements. Ocular artefacts were removed by source separation using Independent Component Analysis (ICA) [37] using eye movements recorded by the EOG channel. Artefacts related to movement or noise from unknown sources were removed by removing EEG segments. Then, all channels were referenced to the Cz channel.

**Figure 2:**
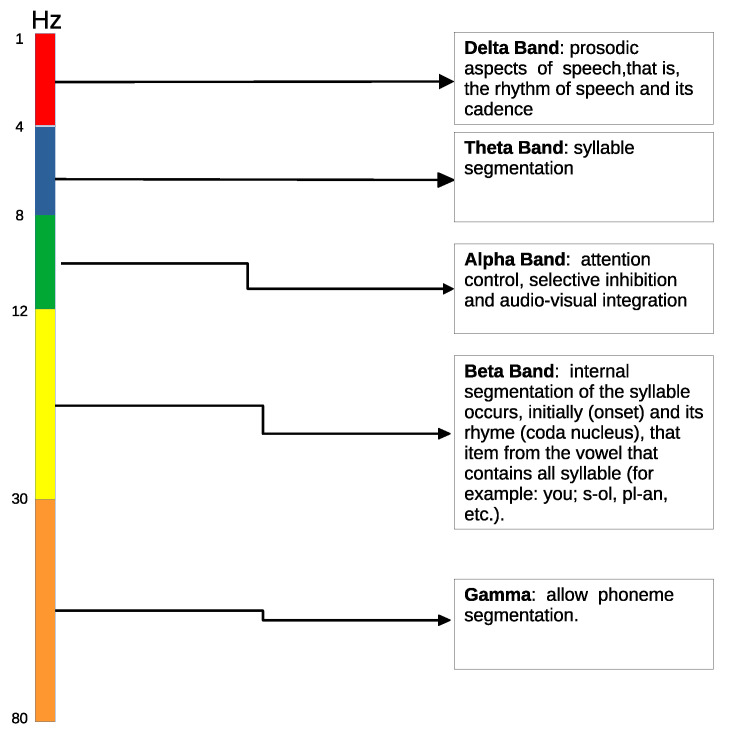
Five main electrophysiological (EEG) frequency bands [39].

After this first stage, the EEG channels were band-pass filtered to extract the information corresponding to the five EEG frequency bands (Delta, 1.5–4 Hz; Theta, 4–8Hz, Alpha, 8–13 Hz; Beta, 13–30 Hz; and Gamma, 30–80 Hz), as shown in Figure 2. It is worth noting that, since we are interested in the phase of the signals, the filtering process cannot introduce phase distortion. This way, Infinite Impulse Response (IIR) filters cannot be used. Instead, we used Finite Impulse Response (FIR) filters, which introduce a constant phase lag that can be corrected afterwards. More specifically, we used a two-way zero-phase lag band-pass FIR Least-Squares filter, which compensates the phase lag introduced while filtering by passing the signal forward and backward through the filter, achieving zero-lag phase in the overall filtering process [38]. Low-pass filtering was applied with a cut-off frequency of 80 Hz. Additionally, a 50 Hz notch filter was used in the preprocessing stage to remove this frequency component.

## 3. Functional Connectivity from EEG Signals

In this section, we show the method used to use the phase difference between EEG channels to infer connectivity, as well as the overall proposed method for classification.

### 3.1. Phase-Based Connectivity

One way to estimate the connectivity between two channels consists of analysing the phase difference of these channels. This has led to the development of different measures for *phase coherence* related to the synchronization of these channels. Since the phase of the signal extracted from an electrode varies over time, it is necessary to compute the phase, $\phi_{i}\left(t\right)$ for each channel *i*, namely the instantaneous phase. In this work, the instantaneous phase is obtained by means of the Hilbert transform computed from band-pass filtered signals.

#### Hilbert Filter

Hilbert transform (HT) allows to compute the analytic signal from a real one. The analytic signal is a complex-valued time series which has no negative frequency components. Thus, it is possible to compute the time varying amplitude, phase and frequency from the analytic signal, also called instantaneous amplitude, phase and frequency, respectively. Hilbert Transform (HT) is defined for a signal x(t).

(1) $$H\left[x\left(t\right)\right]=\frac{1}{\pi }\int_{-\infty }^{+\infty }\frac{x(t)}{t-\tau }d\tau$$

and the analytic signal $z_{i}\left(t\right)$ for a signal x(t) can be obtained as

(2) $$z_{i}\left(t\right)=x_{i}\left(t\right)+jH\left\{x_{i}\left(t\right)\right\}=a\left(t\right)e^{j\phi (t)}$$

From $z_{i}\left(t\right)$, it is straightforward to compute the instantaneous amplitude as

(3) $$a\left(t\right)=\sqrt{re{\left(z_{i}\left(t\right)\right)}^{2}+im{\left(z_{i}\left(t\right)\right)}^{2}}$$

and the instantaneous, unwrapped phase is

(4) $$\phi \left(t\right)=tan^{-1}\frac{im(z_{i}\left(t\right))}{re(z_{i}\left(t\right))}$$

This way, the use of the previous explained method provide the phase value at each time point, allowing to estimate synchronization between channels from phase differences. HT is found useful in the characterization of the EEG through the synchrony of its channels. For instance, in [40] it is used to detect changes of the phase synchronization in epileptic subjects. They found evidences that epileptic seizures can be preceded by characteristic changes in synchronization. In [41], HT is part of a two steps process called empirical mode decomposition [42]. The authors used this process as a feature extractor that is then employed to classify various sleep stages.

The implementation of this method can be found in Scipy [43] library.

### 3.2. Channel Synchronization by Pearson’s Circular Correlation

One method to estimate the connectivity between channels consists of measuring phase difference between EEG channels. One of the most popular phase-based connectivity measures that estimates the extent of synchronization between channels is the Phase Locking Value (PLV). PLV measures the average of phase differences between channels over time [44], and can be computed from the Hilbert-filtered signal, using the instantaneous phase values as:

(5) $$PLV=\left|\frac{1}{n}\sum_{t=0}^{n}e^{j(\phi_{xt}-\phi_{yt})}\right|\;$$

where $\phi_{xt}$ and $\phi_{yt}$ are the phase angle at time point *t* for the channels *x* and *y*, respectively, and *n* is the number of samples of the signal.

Phase-based connectivity measures that relies on the phase difference have advantages over other synchronization measures. One of the most important is the ability to detect false connectivity due to volume conduction. This consists of capturing signal from the same source by two neighbouring electrodes, due to the spreading effect through the skull. Since PLV values due to volume conduction are clustered around zero, it will reflect a zero value in the connectivity matrix, indicating the absence of connection between the corresponding channels. However, PLV is a measure of the consistence of phase difference (average) but it does not imply information exchange between channels which would imply covariance, and could eventually provide spurious connections [45]. To overcome this, in this work, we used Circular Correlation to estimate the phase synchrony. Coherence measures have some limitations as indicated in [44]: on the one hand, since coherence is a measure of the linear covariance between two-spectra, it is not recommended for non-stationary signals. On the contrary, phase-locking based measures do not require the signals to be stationary. On the other hand, coherence increases with amplitude covariance which could bias the estimation of the between channels interaction measurement.

As explained in [45], Circular Correlation measures how the phase variance of two channels co-varies: to determine whether one channel is slightly in advance of its expected phase at a given time, the phase in the other channel is also advanced (in this example, we refer to positive correlation). In the case of unrelated channels, the phase variance will not co-vary and the Circular Correlation will be zero. On the contrary, as the PLV only measures the phase difference, it is likely to be poorer at discriminating between related and unrelated signals [44].

Since phase information is circular (in the range [0,2π]), we use circular statistics [46]. Specifically, the circular equivalent of Pearson’s correlation coefficient [47] can be defined as:

(6) $$r_{circular}=\frac{\sum_{t=1}^{n}\left[sin\left(\phi_{xt}-\bar{\phi_{x}}\right)sin(\phi_{yt}-\bar{\phi_{y}}\right]}{\sqrt{\sum_{t=1}^{n}sin{\left(\phi_{xt}-\bar{\phi_{x}}\right)}^{2}}\sqrt{\sum_{t=1}^{n}sin{\left(\phi_{yt}-\bar{\phi_{y}}\right)}^{2}}}$$

where $\phi_{xt}$, $\phi_{yt}$ is the phase value of the channels *x* and *y* at time point *t*, $\bar{\phi_{x}}$, $\bar{\phi_{y}}$ the circular mean of *x* and *y* channels, and *n* the number of samples of the signal. The circular mean of a channel *i* can be computed as:

(7) $$\bar{\phi_{i}}=arctan\left(\frac{\sum_{t=1}^{n}sin\left(\phi_{it}\right)}{\sum_{t=1}^{n}cos\left(\phi_{it}\right)}\right)$$

According to the above definitions, $r_{circular}$ measures the circular covariance of differences between the observed phase and the expected ($\bar{\phi_{i}}$) phase. As a result, relationship (i.e., synchronization) between channels is detected as a co-variation between the phase variance of the channels. On the other hand, out-of-sync channels will not co-vary and the circular covariance will take values close to zero. In the case of Pearson’s correlation, $r_{circular}$ will take values in the range [0, 1].

## 4. Diagnosing Dyslexia by Outlier Detection

The most usual way to deal with the classification of instances of a dataset, consists of obtaining a discriminative model of the classes. However, this is not always possible for biomedical data: firstly, it is difficult to acquire a balanced dataset, according to the prevalence of the disorder under study. Moreover, biomedical data acquisition is time-consuming and requires a careful screening process. On the other hand, depending on the experimental setup, the nature of the biomedical signals and the objective of the experiment, one (or both) groups under study may contain a considerable variability. These issues can generate a biased model, complicating the generalization capabilities. An effective solution to this situation is using an outlier detection method, modelling the most numerous class and identifying samples belonging to a different class as an outlier (or *anomaly* sample). In this work, we propose the use of an outlier detection method based on self-organizing map, which is trained with the aforementioned circular correlation features.

### 4.1. Outlier Detection Based on Self-Organizing Maps

Self-Organizing Map (SOM) is a vector quantization algorithm that provides a low dimensional projection of a high dimensional feature space, facilitating the visualization of the data structure as well as the easy identification of clusters in data. Works with SOM have been proposed in a wide range of different fields. In medicine [48], SOM was used to group molecular leukaemia samples using the genes as features. It correctly clustered two types of cancer cells. In the computer vision field, Lawrence et al. [49] applied the SOM to reduce the dimensionality of a image at a first step of a pipeline for face recognition. Betti et al. [50] designed a fault prediction system in large power plants based on SOM. An improved SOM algorithm was used in the work of Cai et al. [51]. They employed SOM to classify different sitting postures. In [52], more general experiments were carried out in order to first the dimensionality of the data and then cluster it. Their results show that it is worth spending time training a SOM before attempting to cluster the data. Other fields such as network security have also taken advantage of the properties of SOMs. For instance, in [53], a SOM-based method allows computing bayesian activation of SOM units to detect anomalies in computer networks.

We use the minicom [54] package for python to work with SOM.

The SOM algorithm is briefly explained as follows. Let X $\in R^{n}$ be a *n*-dimensional dataset. The SOM map is composed of *d* units, arranged in a 2-dimensional lattice and each represented by a n-dimensional model vector $\omega_{i}$. For each input data instance x, the Best Matching Unit (BMU) is defined as the unit $\omega_{i}$ closest to x in terms of the euclidean distance ∥·∥:

(8) $$∥\omega_{i}-x∥\leq ∥\omega_{j}-x∥,\forall x\in X,i\neq j$$

Model vectors in a neighbourhood of the BMU are updated in each iteration, according to the euclidean distance to the input data sample.

(9) $$\omega_{i}\left(t+1\right)=\omega_{i}\left(t\right)+\alpha \left(t\right)h_{i}\left(t\right)\left(v-\omega_{i}\left(t\right)\right)$$

where α(t) is the learning rate and $h_{i}\left(t\right)$ is a function which defines the neighbourhood around the BMU $\omega_{i}$, and *i* is a linear index that identify the prototype vectors.

The usual way to progressively reducing α(t) with the number of iterations consist of applying an exponential decay rule [55]. Similarly, the neighbourhood of each SOM unit $h_{i}$ is a Gaussian hat [55] whose width shrinks in time (iterations). In order to improve the convergence of the SOM, it has been linearly initialized to the principal components of the training data. This way, each dimension of prototypes was arranged proportionally corresponding principal component [55].

According to the SOM definition shown above, a data sample *x* will be represented by the prototype vector of the corresponding BMU. The representation error, known as quantization error (QE), can be computed as for each BMU as

(10) $$qe=\frac{1}{n}\sum_{k=1}^{n}∥\omega_{i}-x_{k}∥\;\;\forall x_{k}\in RF_{i}$$

where $RF_{i}$ is the receptive field of the BMU unit $\omega_{i}$, defined as:

(11) $$RF_{i}=\{x_{k}\in X:\|x_{k}-\omega_{i}\left\|\leq \right\|x_{k}-\omega_{j}\left\|\forall i\neq j\right\}$$

#### 4.1.1. Band Relevance Using Quantization Error Distribution

**Figure 3:**
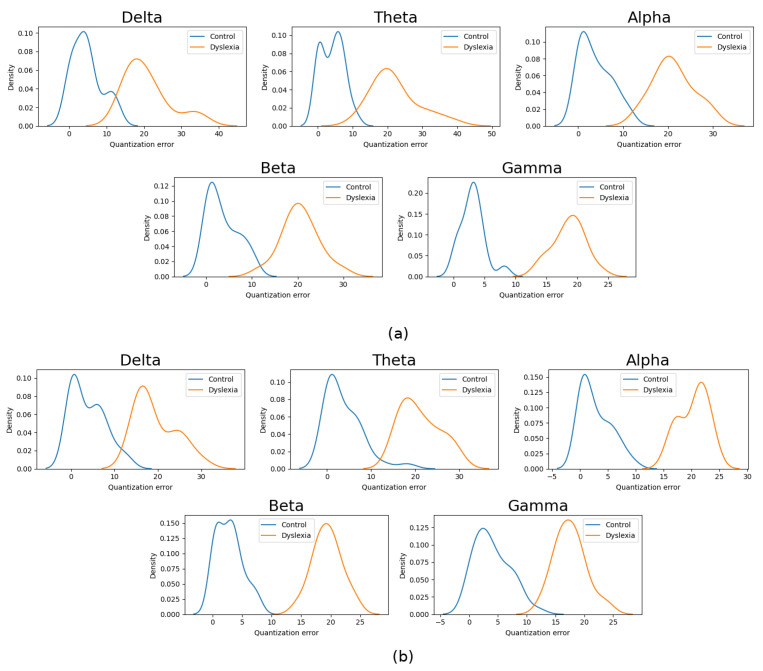
Quantization error distribution for Controls and Dyslexia subjects. The (**a**) 4.8 Hz and (**b**) 16 Hz stimulus.

QE distribution for Controls and Dyslexia subject can be computed using the values provided by Equation (10). Figure 3 shows the distribution of the QE obtained for each EEG band, clearly showing the discriminant capability, as it is possible to find a threshold in QE to separate QE values provided by samples of different classes. Moreover, it is possible to take advantage of this result to select the most discriminant band, since overlapping between distributions can be seen as a measure of separability. In this case, the Kullback–Leibler divergence ($D_{KL}$) [56], defined as:

(12) $$D_{KL}\left(P|Q\right)=\sum_{i}P\left(i\right)\frac{P(i)}{Q(i)}$$

is used to quantify the overlapping between *P* and *Q* distributions and eventually, the discriminative capability of the corresponding error quantization for each data sample. Although $D_{KL}$ is not a symmetric measure and cannot be interpreted as a distance, it is possible to symmetrise by computing a two-ways measure as ${\hat{D}}_{KL}=0.5*\left(D_{KL}\left(P|Q\right)+D_{KL}\left(Q|P\right)\right)$.

According to this measure, $KL_{gamma}>KL_{beta}>KL_{theta}>KL_{alpha}>KL_{delta}$ for 4.8 Hz stimulus and $KL_{alpha}>KL_{beta}>KL_{theta}>KL_{gamma}>KL_{delta}$ for 16 HZ stimulus.

#### 4.1.2. Uncertainly in SOM Units Activation

**Figure 4:**
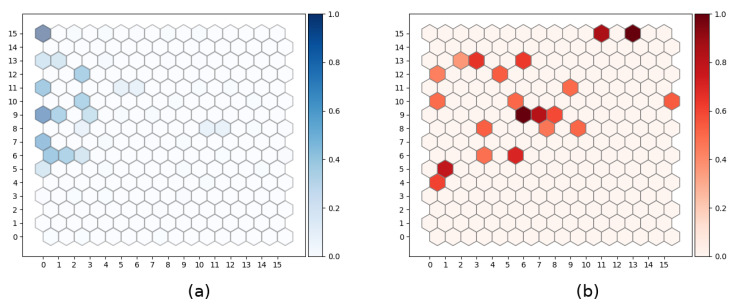
SOM map activation for each data sample, according to the corresponding mean quantization error. Example for EEG Delta band using 4.8 Hz stimulus. (**a**) Control (**b**) Dyslexia.

As explained above, SOM is a vector quantization algorithm that projects the high-dimensional input space into a lower dimensional (usually 2D) lattice. The trained map poses interesting properties such as topology preservation and vector space interpolation between units. Since the BMU unit is computed as the nearest prototype according to the euclidean distance, each data sample will have its BMU, regardless of the resemblance to the map prototypes. However, the reliability of the unit activation can be measured in terms of the quantization error. Thus, the prototype of a BMU producing a large quantization error for a data sample, will barely represent that sample. In Figure 4, the activation of SOM units for all the available samples is shown, according to the corresponding mean quantization error.

As shown in Figure 4, dyslexia samples (considered as anomalies) produces a considerably higher quantization error than control samples.

### 4.2. Bayesian Anomaly Detection for SOM

Naïve Bayes classifiers [57] separate data into different classes by means of the Bayes’ Theorem, along with the assumption of independence among predictors. However, the extent of this premise in the performance of the classifier has been evaluated and discussed in the literature, concluding that it is possible to outperform other classifiers even in the case that all the features are not completely independent [58,59,60]. As a matter of fact, there are different works that take advantage of the benefits of Naïve Bayes classifiers for EEG signal classification. For instance, in [59], a higher number of principal components (PC) used to project the EEG data does not reduce nether the classification accuracy nor the AUC value in comparison to the performance obtained with only one PC, where the feature independence is automatically assumed. On the other hand, Naïve Bayes classifiers provides a good performance with relatively small datasets while avoiding the problems derived from the dimensionality, and without suffering from overfitting. This is especially important with relatively small datasets, where supervised classifiers are easy to overfit, decreasing its generalization capabilities [61].

As shown in the previous section, the anomaly detection method used here is based on the quantization error generated by abnormal patterns in comparison to the one produced by control patterns used to train the map. Since the prototypes only represent control samples, a higher quantization error will be obtained when representing an abnormal pattern using the BMU of the map. Then, it is necessary to compute the threshold for the quantization error to distinguish a normal sample from an anomaly. This threshold can be computed by a Naïve Bayes strategy as follows. Let CN be the *Normal class* and DD the *Anomaly class*. According to Bayes’s theory, the probability of a new sample *x* to belong to the *c* class can be expressed as:

(13) $$P\left(c|x\right)=\frac{P(x|c)P(c)}{P(x)}$$

where P(c) is the prior likelihood of the *c* class, P(x|c) is the probability of *x* conditioned to *c*, and P(x) is the marginal probability, computed as

(14) $$P\left(x\right)=\frac{\#nearest\;samples\;to\;x\;in\;terms\;of\;qe}{\#total\;number\;of\;samples}$$

Thus, it is possible to obtain P(c|x) and the most discriminating threshold can be computed as the value of QE for which P(c|x)=0. The implementation used for the classifier can be found in the sklearn python package [62].

## 5. Results

**Figure 5:**
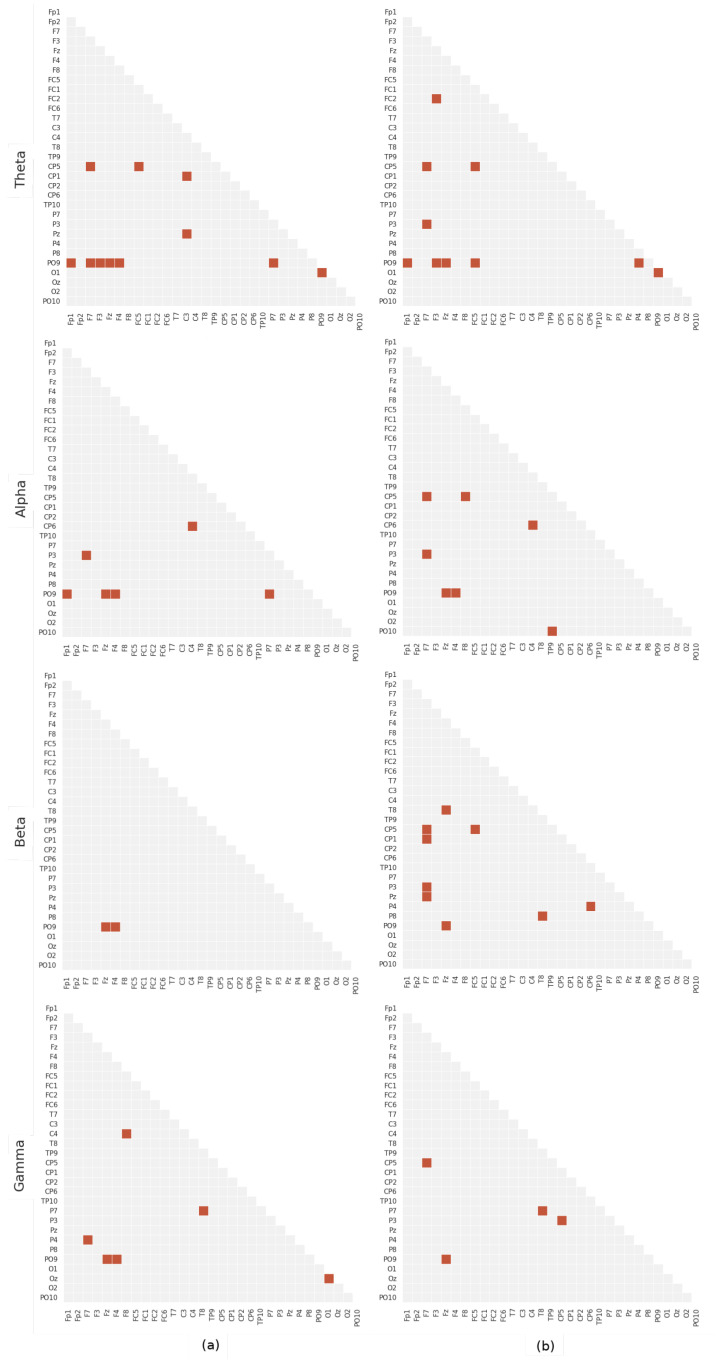
Statistically significant connections for meaningful bands. The axes represent the channels names according to the extended 10–20 EEG montage. Only connections with FDR-corrected p<0.01 are shown. The (**a**) 4.8Hz and (**b**)16 Hz stimulus.

This section presents the main results obtained with the methodology explained above. Since data corresponding to 4.8 and 16 Hz stimuli are stored in the EEG database, we carried out experiments to determine the discriminative power of the different stimuli. As explained in Section 3, we address the searching of differential patterns from a functional connectivity point of view, using the circular correlation as a phase synchrony measure. Thus, in order to show the most significant differences in the connectivity a Mann–Whitney Wilcoxon (distribution agnostic) hypothesis was used. The methodology described at Section 4 to extract features and classify them is applied separately for each EEG band and each stimulus, obtaining the results summarized in Table 2. It is worth noting that this classification methodology is assessed by stratified k-fold cross-validation (k = 5), in order to estimate the generalization errs edit test was performed for each band. Thus, Figure 5 shows the most significant connections as those providing p<0.01 in the hypothesis test. Moreover, in order to remove possible spurious connections, we performed the FDR (False Discovery Rate) correction (alpha = 0.05) of the *p*-values. This also helps to identify significant connections since resultant matrices are sparser.

The methodology described at Section 4 to extract features and classify them is applied separately for each EEG band and each stimulus, obtaining the results summarized in Table 2. It is worth noting that this classification methodology is assessed by stratified k-fold cross-validation (k = 5), in order to estimate the generalization error.

**Figure 6:**
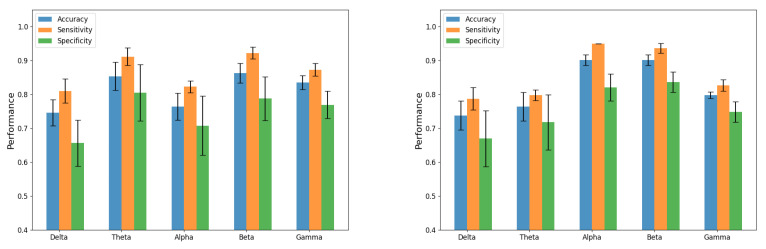
Classification results for 4.8 Hz (**left**) and 16 Hz (**right**).

Additionally, results in Table 2 are graphically depicted in Figure 6, where the error bars corresponds to the standard deviation obtained in the cross-validation process. Specifically, Figure 6 left shows the classification results for the 4.8 Hz stimulus, and Figure 6 right for the 16 Hz stimulus.

**Figure 7:**
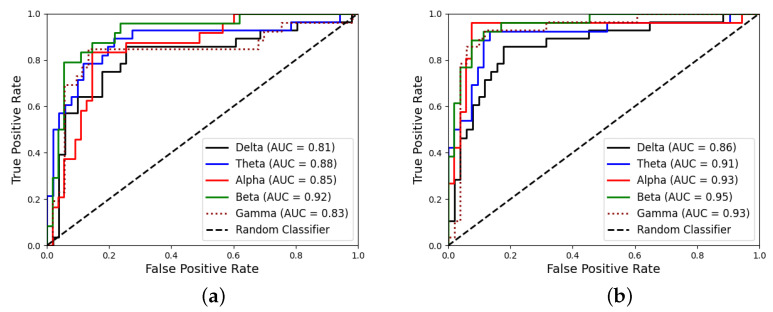
ROC Curves corresponding to the classification results for (**a**) 4.8 Hz and (**b**) 16 Hz stimulus.

It is well known that the overall performance of a binary classifier can be assessed by exploring the Receiver Operating Curve (ROC) space, as it provides the cut-off point of the sensitivity-specificity trade-off. In other words, it shows the performance of the classifier in a compact form, regarding it capability of correctly identifying positive and negative samples. Relatedly, AUC is a very useful metric that represents the probability of a random positive to have a more extreme value of a negative one (and vice versa). Indeed, Figure 7a,b show the ROC curves obtained for each band for the 4.8 Hz and 16 Hz stimulus, respectively, along with the corresponding AUC values computed for each curve.

### Statistical Significance

As usual in biomedical problems, the number of available samples requires performing statistical tests to ensure that the results are not biased by the classification stage (i.e., due to overfitting effects, for instance). In addition, it is necessary to check the probability of these results was obtained by chance, while these tests that can be relatively relaxed when the database is large enough, they require special attention in real-world databases, where classes are not balanced and the sample size is small (in the case of experimental subjects, it is necessary to take into account the prevalence of the disorder being treated. In the case of dyslexia, it is about 7% as indicated in the introduction).

The test used consists on generating a null distribution by calculating the accuracy of the classifier on 1000 permutations of the labels. This provides the distribution for the null hypothesis, which states the independence between features and labels and allows calculating the probability of reproducing the classification results with shuffled labels. This empirical *p*-value is then computed as:

(15) $$p-\mathrm{value}=\frac{\#permutations\;with\;accuracy\;higher\;than\;baseline}{\#Number\;of\;permutations}$$

**Figure 8:**
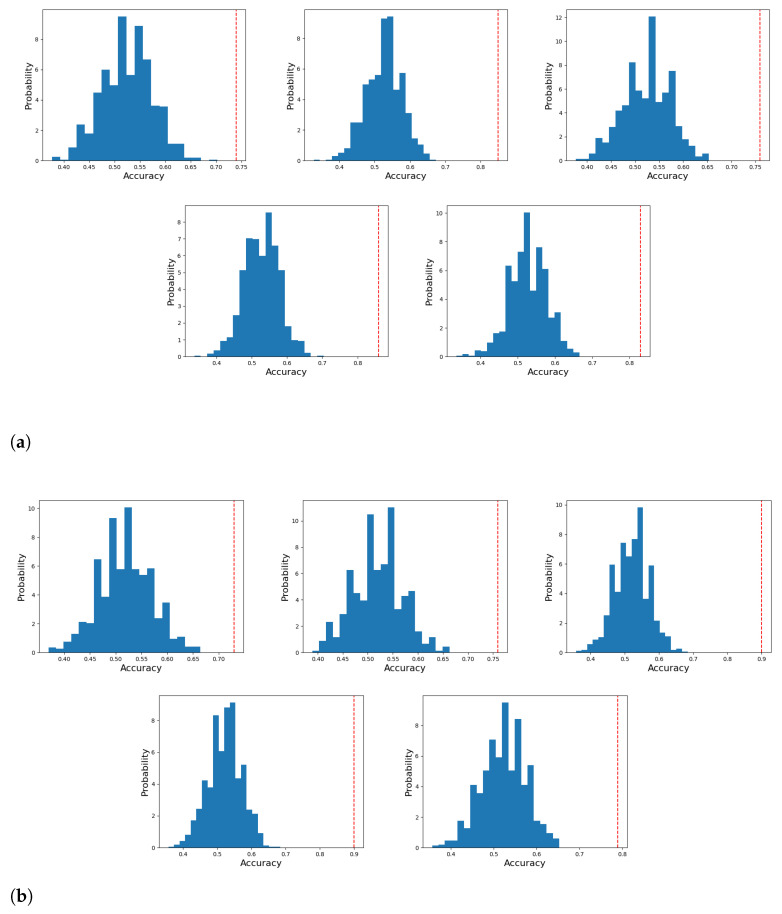
Permutation test results to assess the statistical significance of the results. Blue distribution corresponds to the null distribution and red line corresponds to the accuracy obtained with true labels. The (**a**) 4.8 Hz stimulus and (**b**) 16 Hz stimulus.

The results of the permutation test are depicted in Figure 8, where the null distribution obtained by permuting the labels as indicated above is shown in blue, and the accuracy obtained for the non-permuted case is indicated as a vertical red line. A 5-fold stratified cross-validation is carried out at each permutation iteration. Then, the average of the results obtained at these 5 folds is taken for the corresponding permutation iteration. Thus, Figure 8 shows the probability density for the classification. This is the most computing demanding task as the classifier should be trained 1000 times. In our equipment (Dual Intel(R) Xeon(R) CPU E5-2640 v4 (10 cores)@ 2.40 GHz, 128GB of RAM) it took about one day to complete the permutations.

## 6. Discussion

In this section, discussion about the methodology, results obtained and validation strategy is provided, along with a comparison to previous works addressing the problem of dyslexia classification using biomedical signals.

The proposed method uses the quantization errors corresponding to SOM prototypes as features, which are subsequently used to train a Naïve Bayes classifier that computes the optimum separation threshold. The SOM was trained using all the connections, and the significance of the classification algorithm was assessed by 5-fold cross validation and a permutation test consisting in making 1000 permutations. This ensures the classification results are not obtained by chance, and actual differences are present in patterns belonging to controls and dyslexic subjects.

This validation strategy estimates the generalization error, and bounds the probability of the results to be obtained by chance. With this methodology, Figure 6 also provides a view of the discriminative capabilities of each EEG band. In other words, it can be seen as the influence of each stimulus in the neuronal oscillations at a specific frequency band. Thus, beta and gamma bands are providing the best results for 4.8 Hz stimulus, while the best performance is obtained for Alpha and Beta bands in the case of 16 Hz stimulus. Detailed results in Table 2, show that an accuracy of 0.82 can be obtained with the 4.8 Hz stimulus, along with an AUC of 0.92. The AUC value can be interpreted as the probability of obtaining a higher score for a positive sample than for a negative one, indeed indicating the misclassification probability. In the case of 16 Hz stimulus, the AUC rises up to 0.95, concluding that the most discriminative patterns were found for 16 Hz stimulus in the Beta band.

Moreover, connectivity matrices containing the synchronization measures between each pair of electrodes, compose a connection graph that helps to identify neuroanatomical regions involved in each case. Due to the significance level used and the additional FDR correction, sparse matrices are obtained. This way, the method proposed in this work contributes as a tool to identify synchronized channels that can be related to connections between brain regions. Consequently, the circular correlation used to estimate the phase-synchrony among channels can be effectively used to detect out-of synchrony electrodes, that contribute here to define the outlier (or abnormal sample).

The highest classification performance is obtained for the alpha, beta and gamma bands. The results, as demonstrated later, are highly significant, and therefore we can conclude that there exist connectivity anomalies in the dyslexic brain that the Bayesian classifier can detect. High-frequency patterns (beta, gamma) are relevant for interpreting segment-level representations in the brain [63]; therefore, anomalies in this segmentation would produce cascade effects that would lead into incorrect representation of phonemes in the brain, as suggested by Virtala et al. [64], and inaccurate subsequent predictive processes.

Digging deeper into the matter, Figure 5 depicts the significant inter-channel connections for each stimuli and band. First of all, we find a recurrent interaction between occipital electrodes, such as PO9, PO10, O1, O2 and Oz throughout many bands. These electrodes are very specific to visual processing [65], and could be attributed to differences in visual activity during the auditory stimuli. Many of the other electrodes have been proven to be relevant for current language models [63]. This is the case of the connection between F7/FC5 and P3/CP5, that corresponds to the so-called “dorsal stream” in the Hickock and Poeppel model, that maps acoustic speech signals to frontal lobe articulatory networks. This pattern is present in Theta and Alpha for both stimuli, and Beta/Gamma for the 16 Hz stimulus. The fact that the 16 Hz show additional differences in the Beta/Gamma may be evidence of phonemic-rate stimuli triggering abnormal functioning of higher cognitive tasks.

P3 and CP5 reception fields are both around the temporo-parietal junction, and differences in patterns might be due to differences in placement of the electrodes. However, is fair to assume that they represent the sensorimotor interface in the Hickock and Poeppel model. Similarly, F7 and FC5 might be capturing the activity of the Broca’s area, which is considered an articulatory region in the Hickock and Poeppel model. Occipital-frontal connections such as PO9 to Fz and F4 might be due to the hypothesized conceptual networks, distributed throughout the cortex, that connect lexical and articulatory regions.

On the other hand, the right hemisphere display less, but still relevant anomalies detected by the analysis. There are significant anomalies at the CP6-C4 in the alpha band for both stimuli. wich may correspond to a connection between the right auditory cortex and the right sensorimotor cortex. This deficit has not been found anywhere else. However, it may be coherent with recent research that found atypical rhythmic entrainment in auditory and sensorimotor coupling at low rates [66]. Finally, two interesting differences are found in bilateral connections at the gamma band. First, the P4-F7 anomaly which corresponds to the main result of Molinaro [22] for dyslexia. In that paper, the between the right auditory cortex (P4) Then, the P7-T8 deficit, which involves an anomaly in the connection between the right phonological network (T8) and the right lexical interface on the Hickock and Poeppel model [63].

Regarding the discriminative capability of the proposed method, it has been assessed using the Receiver Operating Curves (ROC), that provide a clearer view of the trade-off between sensitivity and specificity. Hence, ROC curves for all bands are shown in Figure 7 for (a) 4.8 Hz stimulus and (b) 16 Hz stimulus. In both cases a good sensitivity-specificity balance is achieved.

The number of available EEG studies using auditory stimulus for dyslexia diagnosis is limited, and usually focused to exploratory analysis [22,24], in the quest for the biological basis of dyslexia, which is an open research question. Dyslexia classification using biomedical signals is also a current research trend, oppositely to the traditional method based only on behavioural tests. Works using structural imaging [67], MEG [68] and EEG [27,28,29] signals are shown in Table 3. As in [68], the classification problem with biomedical signals is usually addressed using different features extracted from MEG signals, obtaining accuracy values up to 0.93. However, it requires a 255-channel MEG acquisition system. At the same time, MEG data is typically harder to obtain than EEG and more computationally demanding to work with. On the other hand, there are works that use EEG signals for dyslexia classification and obtaining accuracy values up to 0.80 [27]. However, these works usually rely on reading and writing tasks, depending on their own acquired skills and limiting the diagnosis age. The methodology used in this work, based on the analysis of the responses to simple auditory stimuli, overcomes these limitations, makes the test simpler and avoids the possible bias introduced by interactive stimuli or specific task design. On the other hand, the proposed method contributes to the knowledge of the neural basis of dyslexia by showing differential functional connectivity patterns when comparing controls and dyslexic subjects. This has been shown as connectivity matrices to clearly expose the most significant connections. Additionally, our classification results are similar to those obtained with MEG signals, but can be performed with EEG equipment (that can be even portable), facilitating the development of the tasks and their application to young children. This is especially important to our future work continuing this research with pre-readers.

## 7. Conclusions

In this work, we present a method to detect out-of-synchrony EEG channels using cross frequency coupling measures. This measure is derived from the instantaneous phase of the signal acquired from each electrode. In this way, cross frequency coupling techniques provide the way to compute features related to synchronization between electrodes and taking into account the frequency band in which the synchronization takes place. Our proposed method uses the circular correlation of the unwrapped phase to evaluate phase locking between channels, which is further used to construct a connectivity matrix, indicating those electrodes that are synchronized at each frequency band. Differential connectivity patterns demonstrate that low level auditory stimulus generates different interactions between brain areas in controls and dyslexic children, revealing differences in auditory processing from a connectivity point of view. On the other hand, the low SNR of EEG signals and the inherent differences among subjects introduce a high variability among EEG patterns, especially in experimental subjects. This has been addressed by an anomaly detection approach, modelling only patterns from the control subjects to further detect patterns of experimental subjects as outliers. The proposed anomaly detection system is based on vector quantization using a Self-Organizing Map, by means of the quantization error associated with each BMU. This allows to measure differences in the quantization error provided by the prototypes representing control and experimental samples. Additionally, the threshold in the quantization error that optimally separates between the controls and experimental patterns is computed using a Bayesian method to constitute a Naïve Bayes classifier. The results obtained show sensitivity values up to 95% with specificity values up to 83% (AUC up to 0.95), demonstrating the discriminative capabilities of the proposed method to detect out-of-synchrony channels. Thus, it clearly differentiates connectivity patterns from dyslexia subjects from controls, which paves the way to construct an effective and objective computer-aided diagnosis tool.

It is worth noting that this study is based on non-speech, non-interactive stimuli, whose properties are derived from the Spanish language (related to the different language segmentation tasks developed in the brain during language processing). Thus, the study is limited to Spanish speakers and could not be generalized to other languages. On the other hand, the subject sample (7 years old children) in this study were all Spanish speakers from schools of Junta de Andalucía (Andalucía region in the south of Spain). In this way, the extent of the results are limited to the sample diversity.

As a future work, we plan to use a high-density EEG searching for additional low-level interactions among brain areas that could be interesting from an exploratory point of view.

## Figures and Tables

**Table 1:** Database. Age range: 88–100 (t(1)=−1.4,°p>0.05).

Group	Male/Female	Mean Age (Months)	Observations
Control	17/15	94.1±3.3	No reported reading or spelling difficulties
Dyslexia	7/9	95.6±2.9	Formal diagnosis by a clinician expert

**Table 2:** Summary of the best classification results obtained for the different EEG bands. Bands providing the best accuracy values are highlighted in bold.

Stimulus	Band	Accuracy	Sensitivity	Specificity	AUC
4.8 Hz	Delta	0.74 ± 0.03	0.81 ± 0.03	0.65 ± 0.06	0.81 ± 0.05
	Theta	0.85 ± 0.04	0.91 ± 0.03	0.80 ± 0.08	0.88 ± 0.05
	Alpha	0.76 ± 0.04	0.82 ± 0.02	0.70 ± 0.09	0.85 ± 0.08
	**Beta**	**0.86 ± 0.03**	**0.92 ± 0.02**	**0.78 ± 0.06**	**0.92 ± 0.08**
	Gamma	0.83 ± 0.02	0.87 ± 0.02	0.76 ± 0.04	0.83 ± 0.05
16 Hz	Delta	0.73 ± 0.04	0.78 ± 0.03	0.67 ± 0.08	0.86 ± 0.07
	Theta	0.76 ± 0.04	0.79 ± 0.02	0.71 ± 0.08	0.91 ± 0.05
	Alpha	0.90 ± 0.02	0.93 ± 0.01	0.82 ± 0.04	0.93 ± 0.09
	**Beta**	**0.90 ± 0.02**	**0.93 ± 0.02**	**0.86 ± 0.03**	**0.95 ± 0.09**
	Gamma	0.79 ± 0.01	0.82 ± 0.02	0.74 ± 0.03	0.93 ± 0.10

**Table 3:** Classification results for various studies related to dyslexia classification. Standard deviation is indicated in each case. (*) data not reported in the source.

Method	Channels	Acq.Time	Accuracy	Sensitivity	Specificity	AUC
MRI + SVC [67]	T1-MRI	*	0.8 ± *	0.82 ± *	0.78 ± *	*
MEG + SVC + GC [68]	253	3 min	0.63 ± 4.13	0.64 ± 4.01	0.65 ± 4.15	*
MEG + SVC + GE [68]	253	3 min	0.94 ± 1.78	0.93 ± 1.39	0.93 ± 2.32	*
MEG + SVC + CI [68]	253	3 min	0.80 ± 1.14	0.80 ± 1.41	0.79 ± 2.17	*
MEG + SVC + wIFCG [68]	253	3 min	0.97 ± 1.89	0.96 ± 1.89	0.95 ± 1.98	*
EEG + SVC [27] (Writing Task)	32	1 min	0.59 ± *	0.64 ± *	0.53 ± *	*
EEG + SVC [27] (Typing Task)	32	1 min	0.78 ± *	0.88 ± *	0.66 ± *	*
EEG + OCSVC [28]	32	5 min	0.71 ± *	0.53 ± *	0.78 ± *	0.79 ± *
EEG + DAE [30]	32	5 min	0.56 ± *	0.76 ± *	0.66 ± *	0.74 ± *
**Proposed**	**32**	**5 min**	**0.90 ± 0.02**	**0.93 ± 0.02**	**0.86 ± 0.03**	**0.95 ± 0.09**

## Abbreviations

The following abbreviations are used in this manuscript:

EEG	Electroencephalography
DD	Developmental Dyslexia
MEG	Magnetoencephalography
SNR	Signal to Noise Ratio
ICA	Independent Component Analysis
SC	Spectral Coherence
CFC	Cross Frequency Coupling
PAC	Phase Amplitude Coupling
LI	Language Impairment
SSD	Speech Sound Disorder
ADHD	Attention Deficit Hyperactivity Disorder
IIR	Infinite Impulse Response
FIR	Finite Impulse Response
HT	Hilbert Transform
PLV	Phase Locking Value
SOM	Self-Organizing Map
BUM	Best Matching Unit
ROC	Receiver Operating Curves
AUC	Area Under ROC Curve
